# Investigating interferon type I responses in patients with suspected giant cell arteritis and polymyalgia rheumatica

**DOI:** 10.1093/cei/uxae085

**Published:** 2024-10-04

**Authors:** Marieke van Nieuwland, A H Leontine Mulder, Edgar M Colin, Celina Alves, Lenny van Bon, Elisabeth Brouwer

**Affiliations:** Department of Rheumatology and Clinical Immunology, Hospital Group Twente (Ziekenhuisgroep Twente), Almelo, The Netherlands; Department of Rheumatology and Clinical Immunology, University of Groningen, University Medical Center Groningen, Groningen, The Netherlands; Clinical Laboratory, Unilabs Oost, Enschede, The Netherlands; Department of Clinical Chemistry, Hospital Group Twente (Ziekenhuisgroep Twente), Almelo, The Netherlands; Department of Rheumatology and Clinical Immunology, Hospital Group Twente (Ziekenhuisgroep Twente), Almelo, The Netherlands; Department of Rheumatology and Clinical Immunology, Hospital Group Twente (Ziekenhuisgroep Twente), Almelo, The Netherlands; Department of Rheumatology and Clinical Immunology, Hospital Group Twente (Ziekenhuisgroep Twente), Almelo, The Netherlands; Department of Rheumatology, Radboudumc, Nijmegen, The Netherlands; Department of Rheumatology and Clinical Immunology, University of Groningen, University Medical Center Groningen, Groningen, The Netherlands

**Keywords:** vasculitis, chemokines, cytokines, Interferon, polymyalgia rheumatica

## Abstract

Giant cell arteritis (GCA) and polymyalgia rheumatica (PMR) are closely related inflammatory disorders. Easily measurable biomarkers defining active disease and identifying patients in need of glucocorticoid sparing treatment options are highly desired. Interferon Type I (IFN-I) might be involved in disease pathology; however, evidence is limited. This study explores a systemic IFN-I signature and expression of IFN-I markers in GCA and PMR patients. Treatment naive GCA and PMR patients, and PMR patients with glucocorticoid treatment were included. Patients suspected of but not diagnosed with GCA were used as controls. Five relevant IFN-I-stimulated genes were identified in literature, and relative expression levels were determined using quantitative reverse transcription polymerase chain reaction (RT-qPCR) in peripheral blood mononuclear cells. An IFN-I score was generated. Serum levels of IFN-I induced C-X-C motif chemokine 10 (CXCL10) and Galectin-9 were determined by multiplex immunoassay. There were no differences in IFN-I scores between the groups. An IFN-I signature was observed in 0/9 controls, 2/11 GCA patients, 4/20 treatment naive PMR patients, and 2/10 PMR patients with treatment. Serum CXCL10 and Galectin-9 were not increased in GCA or PMR patients compared to control patients. Treated PMR patients had lower CXCL10 levels [423.2 pg/ml (375.1–491.1)] compared to treatment naive PMR patients [641.8 pg/ml (552.8–830.6)]. An IFN-I signature does not distinguish GCA and PMR patients from controls. Also, IFN-I-induced serum markers are not upregulated in GCA and PMR patients. Easily measurable IFN-I-induced serum markers will therefore probably not aid in diagnosis and additional treatment options in newly diagnosed GCA and PMR patients.

## Graphical abstract

**Graphical Abstract:**
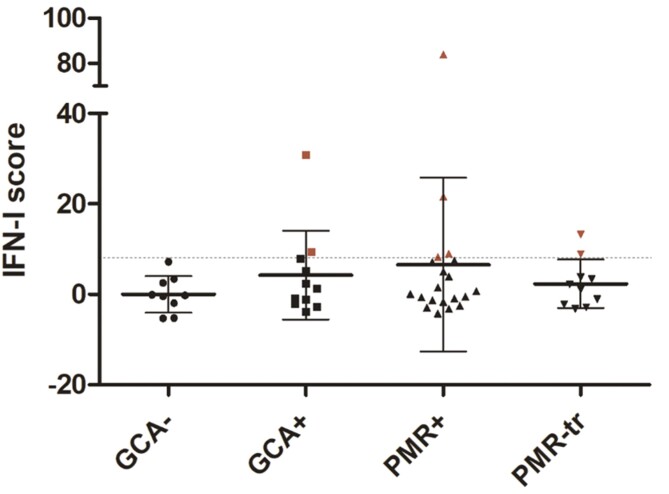

## Introduction

Giant cell arteritis (GCA) and polymyalgia rheumatica (PMR) are closely related rheumatic inflammatory conditions, occurring in patients over 50 years old [1]. GCA affects middle- to large-sized arteries and can lead to severe complications such as permanent blindness and stroke when not appropriately treated [2, 3]. However, recognition of GCA remains challenging due to nonspecific symptoms [4]. Patients with PMR present primarily with (morning) stiffness and/or proximal pain of the shoulder and hip area, leading to a significant decline in quality of life [5]. GCA and PMR often overlap, as in 40–60% of patients with GCA, PMR occurs simultaneously [6, 7]. Furthermore, subclinical GCA can exist in PMR patients [8]. Recently, it is advocated that PMR and GCA should be considered as a spectrum disease rather than two individual conditions [9]. Immunopathology of GCA and PMR is still largely unknown. Increased erythrocyte sedimentation rate (ESR) or increased C-reactive protein (CRP) levels are indicators of GCA and/or PMR; however, these markers are nonspecific. Therefore, the identification of easily measurable biomarkers mainly defining patients with active GCA or PMR is highly desired [10]. Both GCA and PMR are effectively treated with glucocorticoids (GCs). However, severe side effects illustrate the need for GC-sparing personalized treatment options and early identification of patients in need of this is relevant in daily clinical practice [11].

Interferon type I (IFN-I), a group of pro-inflammatory cytokines mainly involved in viral immune responses, plays a role in various systemic autoimmune diseases such as systemic lupus erythematosus and systemic sclerosis [12–14]. Although the hypothesis that GCA and PMR are viral-induced diseases regularly mentioned in literature [15, 16], there is still little evidence for the potential importance of IFN-I signaling in GCA and PMR. A small study by Nordborg *et al*. [17] showed increased expression of myxovirus resistance Protein A (MxA), an IFN-I-induced protein, in temporal arteries of PMR patients and of four GCA patients. Our group supported this finding by showing increased MxA expression in temporal arteries, which indicates a possible role of IFN-I in GCA [18]. Unraveling the role of IFN-I in GCA and PMR may give insight in disease mechanisms and provide a first step into identification of biomarkers. Therefore, it can possibly aid in diagnosis and treatment of these diseases [19].

Although several studies are conducted that describe overexpression of IFN-I stimulated genes (ISGs), termed an IFN-I signature, in autoimmune diseases, a systematic approach to identify ISGs that should be used in the IFN-I signature gene panel is lacking [19–21]. Therefore, as a first step, we conducted a literature study to identify a condense gene panel to be used when testing for IFN-I signatures in rheumatic autoimmune disorders. Using this panel, our study aimed to determine the presence of an IFN-I signature in peripheral blood mononuclear cells (PBMCs) in patients with suspected GCA and PMR by gene expression analysis. Furthermore, we investigated easily measurable IFN-I induced serum markers Galectin-9 and CXCL10 using Luminex immunoassay.

## Materials & methods

### Literature search

A literature search was performed on PubMed to select a gene panel reflecting IFN-I activity. Studies describing ISGs in systemic lupus erythematosus, systemic sclerosis, primary Sjögren’s syndrome, rheumatoid arthritis, and vasculitis were selected. A detailed search strategy is provided in Supplementary File 1. Studies that were not performed in humans, used fewer than 10 samples, were not published in English or did not measure ISG expression were excluded. Each ISG described in the papers was scored individually, reflecting both high expression in IFN-I signatures, good discrimination between diseased and control patients, and its prevalence in IFN-I signatures (Table 1). The five ISGs with the highest score were selected for RT-qPCR analysis in the present study.

**Table 1:** Scoring method of IFN-I stimulated genes in literature

Score	Percentage of patients with an IFN-I signature	Percentage of controls with an IFN-I signature	Statistical significance of individual genes
3	≥80%		
2	65–80%		*P* < 0.001
1	50–65%		*P* < 0.01
0	25–50%		*P* < 0.05
–1	1–25%	25–50%	*P* > 0.05
–2	0%	≥50%	

Genes were scored individually based on the percentage of patients and controls that had a positive IFN-I signature in gene panels used in the papers. For papers that only measured individual gene expression, *P*-values between patients and controls were used for the scoring.

### Study design

Patients, both male and female, with suspected GCA or PMR were included consecutively from Hospital Group Twente at first visit between September 2021 and May 2023. Furthermore, patients diagnosed with new PMR who already received GC treatment for a maximum of 4 weeks at time of blood withdrawal were included. Therefore, patients were stratified into four subgroups: (i) control patients who were suspected of GCA but ultimately not diagnosed with GCA, (ii) treatment naive GCA patients, (iii) treatment naive PMR patients, and (iv) PMR patients with GC treatment. Patients were only included when blood analysis was needed for standard care. Patients were excluded from inclusion when they were <50 years old. Patients with GCA or PMR were followed for 6 months, and the diagnosis of the treating physician after 6 months was used as a reference standard. Control patients were referred back to the general practitioner after diagnostic work-up ruled out GCA and were therefore not followed for 6 months. This study was compliant to the Declaration of Helsinki, approved by the local ethics committee, and written informed consent was obtained from all study participants.

### Sample processing

Blood was collected in clotting tubes for serum extraction and heparin tubes for isolation of PBMCs [Greiner Bio-One (GmbH), Kremsmünster, Austria]. Samples were processed the same day as blood collection. Clotting tubes were centrifuged and cell-free serum was extracted. PBMCs were isolated using Sepmate tubes (Stemcell Technologies, Vancouver, Canada) as described in detail elsewhere [22]. Serum samples and PBMCs were stored at –80°C until further use. The expression levels of the five selected ISGs were determined by RT-qPCR. In short, RNA was extracted, and cDNA was synthesized using defrosted PBMCs with the QIAamp Blood RNA kit (Qiagen, Venlo, The Netherlands). For RT-qPCR analysis, experiments were performed in triplicates using predesigned primers, and all samples were normalized to the household gene PBGD (Thermo Fischer Scientific, Waltham, USA). Serum multiplex immunoassay using Luminex technology was performed to measure CXCL10 and Galectin-9 levels as described in detail elsewhere [23].

### Statistical analysis

Baseline characteristics were described in mean values with standard deviation (SD) or median with interquartile range (IQR) when appropriate after testing for normality. Relative expression (RE) of the five ISGs was determined by calculation of fold expression change values using the 2^-ΔΔct^ method, normalized to the household gene. Absolute values of serum CXCL10 and Galectin-9 were described. A one-way ANOVA or Kruskal-Wallis test was performed to compare multiple groups to each other depending on normal distribution. An independent samples *T*-test or Mann-Whitney *U* test was performed when appropriate to compare two groups. A *P*-value of <0.05 was considered as statistically significant. To describe the presence of an IFN-I signature, an IFN-I score was calculated using the sum of the individual RE of the five ISGs after normalization to the control group (∑(RE_subject_ − Mean_control_)/SD_control_) as described before [24, 25]. An IFN-I signature was considered present when the IFN-I score of the individual study subject was higher than the mean IFN-I score + 2SD of the control group [25]. SPSS (v24, SPSS Inc., Chicago, USA) was used for statistical analyses and GraphPad Prism (v5, GraphPad Software Inc., La jolla, CA, USA) for data visualization.

## Results

### Five ISGs were identified in a literature study

In total, 32 papers were included and ISGs used in the IFN-I signatures in these papers were scored individually (see Supplementary File 1 for details). In total, 72 ISGs were identified in this literature search. The median score given to individual genes adhering to Table 1 was a score of 2 (IQR 2–6). The five genes with the highest scores were identified. Based on these scores, widely used *IFI44L*, *MxA*, *IFI44*, *IFIT1*, and *RSAD2* with scores of 26, 31, 31, 33, and 53, respectively, were used for RT-qPCR analyses in the present study.

### Patient characteristics

Based on diagnosis by the treating physician after 6 months of follow-up, 11 patients diagnosed with GCA, 20 treatment naive PMR patients, and 10 PMR patients who received short-term GC treatment were included for analyses. Treatment naive patients were included at baseline visit, before start of GC treatment. Furthermore, nine patients suspected of but ultimately not diagnosed with GCA were included as controls. Mean age was 68.9 (SD 9.5) years and 54.0% (*n* = 27) of patients were female in the total population. There was a statistically significant increase in inflammatory markers ESR and CRP for all disease groups compared to the control group (see Table 2 for more detailed baseline characteristics). Out of 11 GCA patients, 10 fulfilled the 2022 ACR/EULAR GCA classification criteria [26]. Out of 20 PMR patients, 18 fulfilled the ACR/EULAR 2012 classification criteria for PMR [27]. All 10 PMR patients who already received GC treatment fulfilled these criteria at their baseline visit. These patients received 15 mg/day (*n* = 9) or 20 mg/day (*n* = 1) GC treatment no longer than 4 weeks at time of blood withdrawal. For all PMR-tr patients, ESR and CRP were (nearly) normalized at time of blood withdrawal compared to first visit with a median ESR of 15 mm/h (IQR 6.5–23.0) and a median CRP of 5 mg/l (IQR 1.5–7.3). Only four patients were clinically in remission at time of blood withdrawal (Supplementary Figure 1).

**Table 2:** patient characteristics at baseline visit

	Controls (*n* = 9)	GCA (*n* = 11)	Treatment naive PMR (*n* = 20)	PMR receiving treatment (*n* = 10)
Age in years; mean (SD)	70.2 (8.8)	75.5 (7.9)	66.8 (9.1)	64.8 (9.7)
Gender; % female (*n*)	77.8 (7)	72.7 (8)	35.0 (7)^a^	50.0 (5)
CRP (mg/L); median (IQR)	2.0 (1.5–7.5)	40.0 (27.0–74.0)^a^	37.0 (12.8–98.3)^a^	32.5 (16.8–46.0)^a^
ESR (mm/h); median (IQR)	10.0 (7.5–25.0)	71.0 (45.0–78.0)^a^	31.5 (26.8–65.5)^a^	39.0 (25.5–64.0)^a^
Cranial symptoms; % yes (*n*)	88.9 (8)	72.7 (8)	5.0 (1)^a^	30.0 (3)^a^
Constitutional symptoms; % yes (*n*)	44.4 (4)	81.8 (9)	55.0 (11)	70.0 (7)

^a^Statistically significant difference compared to control group (*P* < 0.05). Cranial symptoms = new headache, jaw claudication, scalp tenderness, or vision loss. Constitutional symptoms = fever, fatigue, or weight loss. GCA = giant cell arteritis; PMR = polymyalgia rheumatica.

### Mean MxA gene-fold expression change was lower in PMR patients receiving treatment compared to control patients

Table 3 shows mean gene-fold expression change (2^-ΔΔCt^) values of the five ISGs. Looking at differences in individual gene expression between the three disease groups and the control group, only mean MxA RE was significantly lower in PMR patients with treatment compared to control patients (*P* < 0.041). In contrast, no significant differences in MxA expression were observed between control patients and treatment naive GCA or PMR patients. This statistically significant difference between control patients and PMR patients with treatment was not observed for the other four ISGs.

**Table 3:** mean fold changes (2^^-ΔΔCt^) with SD of the five ISGs in PMR and GCA suspected patients

	Controls (*n* = 9)	GCA (*n* = 11)	Treatment naive PMR (*n* = 20)	PMR receiving treatment (*n* = 10)
IFI44L	1.820 (1.7)	1.646 (1.3)	4.255 (9.1)	0.675 (0.5)
IFI44	1.367 (0.9)	1.320 (0.9)	2.420 (5.0)	1.037 (1.3)
IFIT1	1.399 (1.0)	4.635 (7.2)	4.097 (5.4)	0.652 (0.3)
MxA	1.943 (2.5)	2.049 (1.9)	1.648 (1.8)	0.323 (0.4)^a^
RSAD2	1.223 (0.8)	2.101 (1.4)	2.284 (2.7)	4.971 (4.8)

^a^Statistically significant difference with the control group. GCA = giant cell arteritis; PMR = polymyalgia rheumatica.

### An IFN-I signature is not present in the majority of GCA and PMR patients

**Figure 1:**
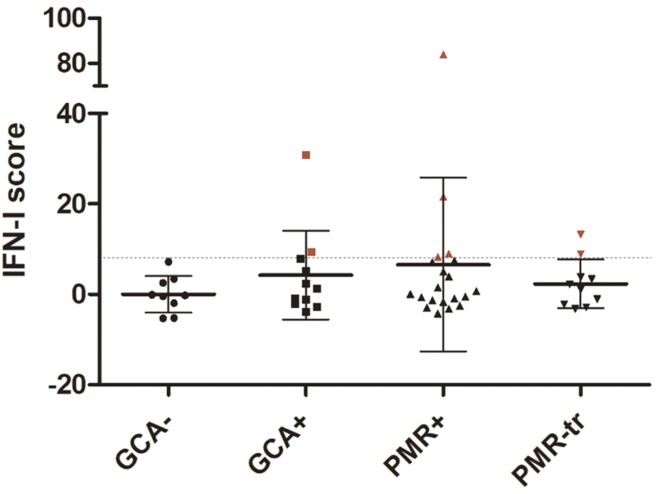
IFN scores (∑(RE_subject_—Mean_control_)/SD_control_) for each individual study subject for control patients (GCA–), GCA patients (GCA+), treatment naive PMR patients (PMR+), and PMR patients receiving treatment (PMR-tr). Patients with an IFN-I signature are depicted in red. Horizontal bars indicate the mean values with SD for each group. The dotted line represents the cut-off value of 8.0 (Mean_control_ + 2SD)

Figure 1 shows the IFN-I scores for each individual study subject. There were no statistically significant differences in IFN-I scores between any of the disease groups compared to the control group. Patients were considered to have an IFN-I signature if their IFN-I score (∑(RE_subject_ –  − Mean_hc_)/SD_hc_) was 2SD above the mean IFN-I score of the control group. Using this, the cut-off value was an IFN-I score of 8.0. In the control group, an IFN-I signature was observed in 0/9 patients. An IFN-I signature was only observed in 2/11 GCA patients, 4/20 treatment naive PMR patients, and 2/10 PMR patients with treatment (Fig. 1). The two PMR patients with treatment who had an IFN-I signature were clinically in remission at time of blood withdrawal with normalized ESR and CRP.

### Serum immunoassay shows no elevated levels of IFN-I induced CXCL10 and Galectin-9 in GCA and PMR patients

**Figure 2:**
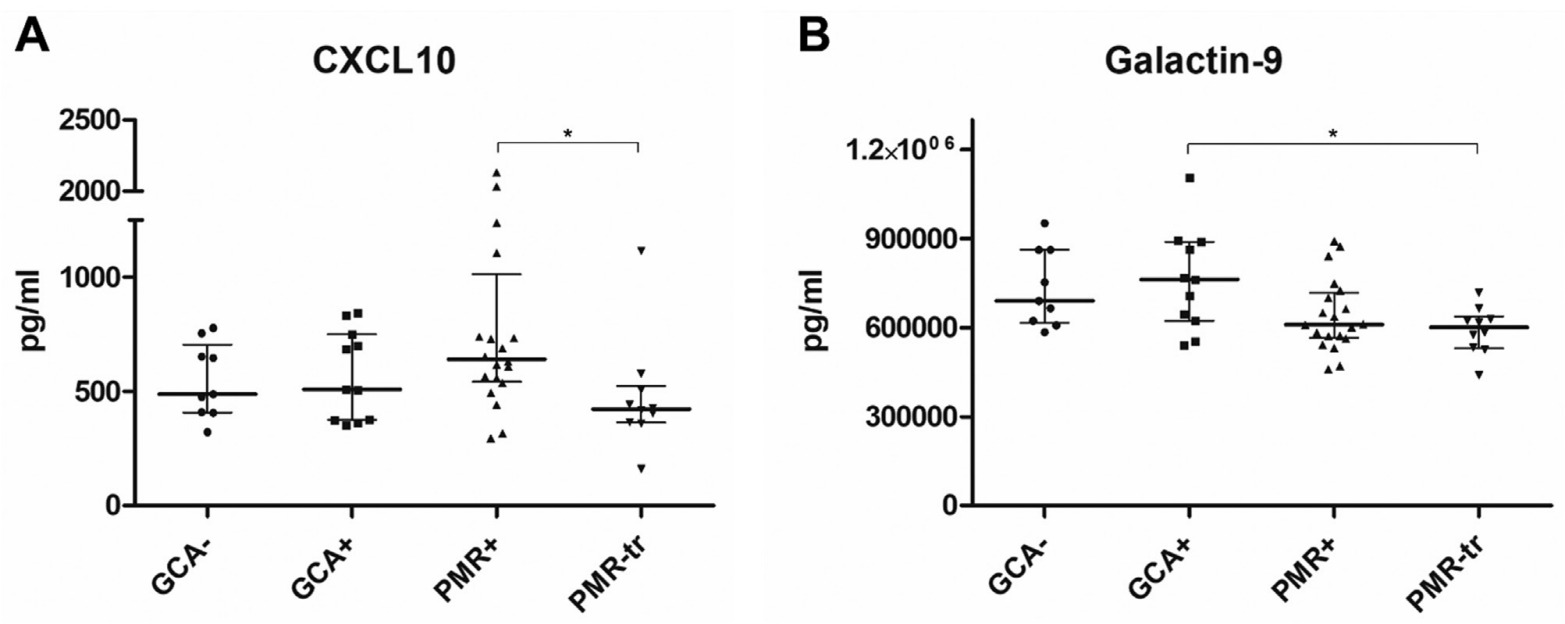
(**A**) Serum CXCL10 and (**B**) Galectin-9 levels for each individual study subject for control patients (GCA–), GCA patients (GCA+), treatment naive PMR patients (PMR+), and PMR patients receiving treatment (PMR-tr). * = *P* < 0.05. Horizontal bars indicate the median values for each group with IQR

In addition to relative ISG expression, presence of IFN-I induced serum markers CXCL10 and Galectin-9 was assessed to validate qPCR findings. Figure 2 shows serum levels of CXCL10 and Galectin-9 for each individual study subject. No statistically significant differences between the control group and disease groups were observed for both markers. For CXCL10, two PMR patients had very high CXCL10 levels compared to other patients. These patients both had an IFN-I signature, and levels of Galectin-9 were higher than the IQR of the PMR group. Median CXCL10 for the control group was 548.0 pg/ml (IQR 408.8–653.1). For the GCA group, this was 508.3 (552.8–725.2); for the treatment naive PMR group, this was 641.8 (552.8–830.6); and for the PMR group with treatment this was 423.2 (375.1–491.1). Median Galectin-9 was 689.5 ng/ml (IQR 622.4–861.7) for the control group, 762.3 ng/ml (633.7–876.2) for the GCA group, 609.6 ng/ml (569.2–705.8) for the treatment naive PMR group, and 600.2 ng/ml (543.1–626.5) for the PMR group with treatment. Both markers seemed to have lower expression levels in PMR patients with treatment compared to the other three study groups. There was a statistically significant difference in CXLC10 expression between treatment naive PMR patients and PMR patients with treatment (*P* < 0.05). Also, PMR patients with treatment had significantly lower expression levels of Galectin-9 compared to the GCA group (*P* < 0.05) (Fig. 2).

## Discussion

This is the first study to explore a systemic IFN-I signature using IFN-I-induced gene expression analysis in GCA and PMR patients. Our data show no difference in ISG expression scores of five selected ISGs between GCA or PMR patients and controls. An IFN-I signature is rarely present in GCA and PMR patients and does therefore not sensitively distinguish GCA and PMR patients from controls. Also, no upregulation of IFN-I-induced serum markers CXCL10 and Galectin-9 in GCA and PMR patients is observed.

Our group previously provided evidence for increased IFN-I activity in GCA patients, as IFN-I-induced MxA was detected in GCA-affected temporal artery biopsies [18]. Another study in patients with aortitis showed an IFN-I signature in inflamed aorta tissue based on transcriptomic analysis [28]. Therefore, IFN-I might play a role in disease pathology locally in the affected artery at disease initiation. Literature suggests that a viral trigger such as a Varicella Zoster infection might be responsible for GCA initiation as it was discovered in temporal artery biopsies of GCA patients [29]. This indicates a role for IFN-I in GCA or PMR pathogenesis. However, two other studies debated the role of Varicella Zoster in GCA initiation even though they also do not exclude it [30, 31]. Our data show no signs of systemic IFN-I activation and thus do not necessarily support a viral trigger. CXCL10 expression was significantly decreased in PMR patients with GC treatment compared to treatment naive PMR patients and also IFN-I scores seemed lower compared to the PMR group, albeit not statistically significant. This was not surprising as GC treatment has strong and quick anti-inflammatory responses [32, 33]. There were only a few patients who showed an IFN-I signature in GCA and PMR patients. These patients did not present with distinct clinical features in comparison to GCA and PMR patients without an IFN-I signature.

A major strength of this study is that we used patients with suspected GCA as a control patients instead of using healthy controls. Disease controls better reflect clinical practice and are therefore more useful to investigate if IFN-I expression could be used as a potential diagnostic marker. Possibly, when healthy controls would be used, a different outcome may have been observed. Furthermore, patients were included before initiation of treatment, and we included a small group of PMR patients who already started treatment for a maximum of 4 weeks as a separate disease group. Our study also has some limitations. First, as this was a pilot study in a rare disease, patient numbers are small. Nevertheless, we expect that this is a valid sample of the affected population to investigate the usefulness of IFN-I in clinical practice. In addition, we only measured five ISGs, but IFN-I-induced serum marker immunoassays confirm our RT-qPCR findings. Furthermore, we identified the five ISGs used in this study by prior literature research. Of note, IFN-I is predominantly studied in SLE. The IFN pathway is a complex system with diverse downstream effects [34]. The 2022 EULAR points for measurements, reporting and application of the IFN-I pathway activation assays in clinical research and practice were considered in this study, but the entire complexity of the IFN-I pathway was not investigated [34]. Therefore, cell-type-specific analysis could identify a minor role for IFN-I in specific disease processes when looking in more detail at specific types of lymphocytes or monocytes. Nevertheless, this was the first study to investigate IFN-I for its clinical relevance at diagnosis and potential monitoring. Validation of our results in a preferably larger prospective cohort possibly using transcriptomics is needed to fully comprehend the role of IFN-I in GCA and PMR. Also, to fully comprehend possible pathogenic aspects of IFN-I in GCA and PMR, the use of an age-matched positive control group should be considered for future research.

In conclusion, an IFN-I signature does not sensitively distinguish GCA and PMR patients from control patients in our cohort. Also, IFN-I-induced serum markers are not upregulated in GCA and PMR patients compared to suspected GCA patients that were ultimately not diagnosed with GCA. Therefore, we do not suspect IFN-I to play a major role in GCA and PMR. Also, easily measurable IFN-I induced serum markers will probably not aid in diagnosis and monitoring in new GCA and PMR patients.

## Supplementary data

Supplementary data are available at *Clinical and Experimental Immunology* online.

uxae085_suppl_Supplementary_Materials

## Data Availability

The data that support the findings of this study are available from the corresponding author, M.v.N., upon reasonable request.

## Abbreviations:

CRP: C-reactive protein
ESR: erythrocyte sedimentation rate
GCs: glucocorticoids
GCA: giant cell arteritis
IFN-I: interferon type I
ISGs: IFN-I stimulated genes
MxA: myxovirus resistance protein A
PBMCs: peripheral blood mononuclear cells
PMR: polymyalgia rheumatica
RE: relative expression
SD: standard deviation
