# Supplementary material for: Investigating interferon type I responses in patients with suspected giant cell arteritis and polymyalgia rheumatica
Source: Clin Exp Immunol. 2024 Oct 4;218(3):308–13. doi: 10.1093/cei/uxae085 (PMC11557144; doi:10.1093/cei/uxae085)
Supplement: uxae085_suppl_Supplementary_Materials [file uxae085_suppl_supplementary_materials.docx]

**Supplementary File S1** literature search details

**Literature study**


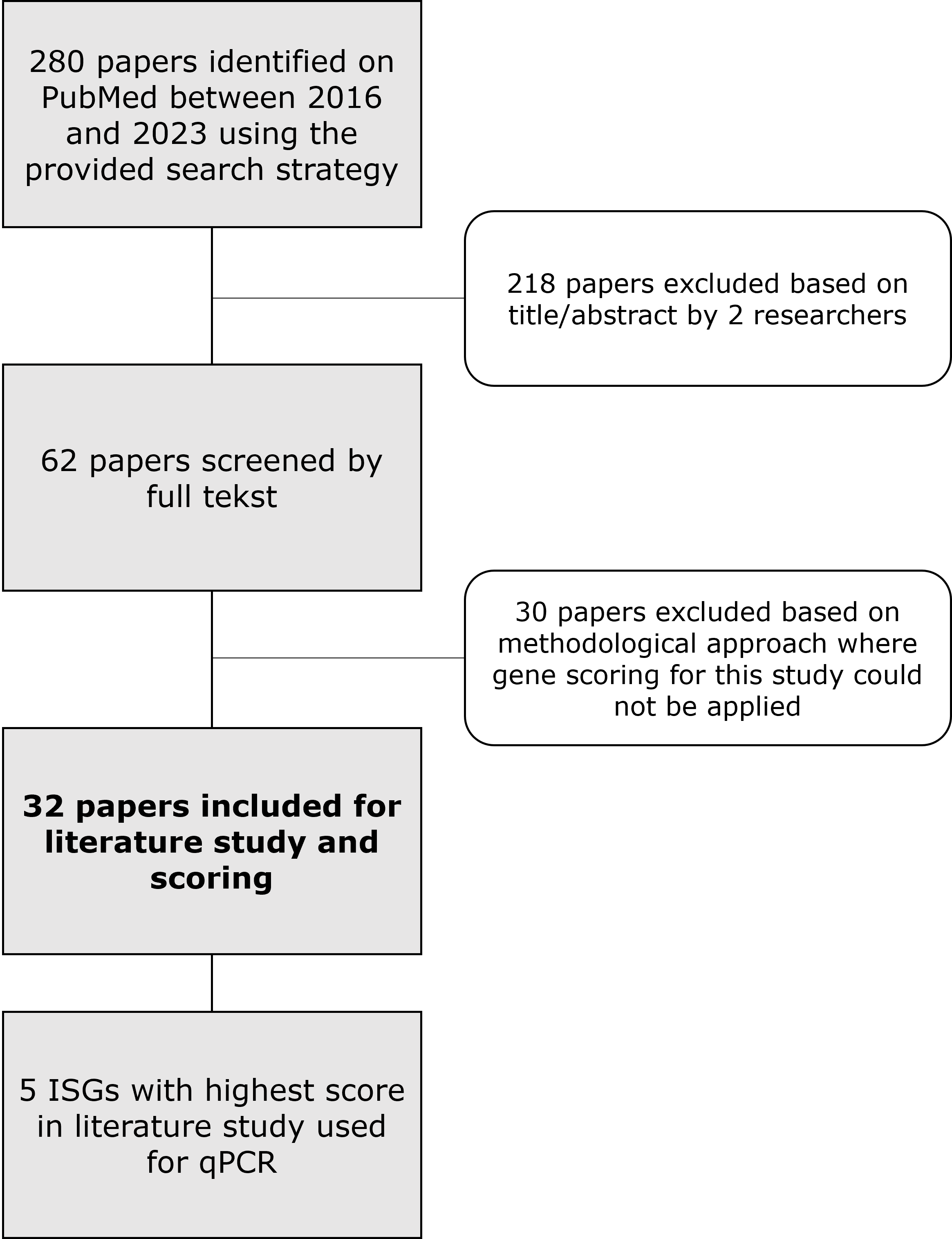
In total, 280 studies were identified between 2016 and 2023 on PubMed. Studies not describing patients with systemic lupus erythematosus, systemic sclerosis, primary Sjögren’s syndrome, rheumatoid arthritis or vasculitis were excluded. Studies that were not performed in humans or were not published in English were excluded. Studies that used fewer than 10 samples or did not measure gene expression using real-time qPCR were also excluded. Genes were scored individually based on the percentage of patients that had a positive interferon type I (IFN-I) signature per study. If this was not described, the difference in gene expression between patients and controls in terms of p-value was used instead (see Table 1; main document). If this score could not be applied due to different methodological approaches, papers were excluded after full text screening.

**Figure 1** Flow chart depicting the process of the paper selection for this literature study to select the 5 genes measured in our study. ISG = interferon stimulated gene

**Search strategy**

(("autoimmune diseases"[MeSH Major Topic] OR "autoimmune"[Title/Abstract] OR "autoinflammatory"[Title/Abstract] OR "inflammaging"[Title/Abstract]) AND "interferon"[Title/Abstract] AND ("signature"[Title/Abstract] OR "interferon regulated genes"[Title/Abstract] OR "interferon stimulated genes"[All Fields]) AND "interferons"[MeSH Terms]) AND (2016:2023[pdat])

**Included studies**

1. Brkic Z, van Bon L, Cossu M, van Helden-Meeuwsen CG, Vonk MC, Knaapen H, et al. The interferon type I signature is present in systemic sclerosis before overt fibrosis and might contribute to its pathogenesis through high BAFF gene expression and high collagen synthesis. Ann Rheum Dis. 2016 Aug;75(8):1567–73.

2. de Jong TD, Lübbers J, Turk S, Vosslamber S, Mantel E, Bontkes HJ, et al. The type I interferon signature in leukocyte subsets from peripheral blood of patients with early arthritis: a major contribution by granulocytes. Arthritis Res Ther. 2016 Jul;18:165.

3. de Jong TD, Vosslamber S, Mantel E, de Ridder S, Wesseling JG, van der Pouw Kraan TCTM, et al. Physiological evidence for diversification of IFNα- and IFNβ-mediated response programs in different autoimmune diseases. Arthritis Res Ther. 2016 Feb;18:49.

4. Ekholm L, Vosslamber S, Tjärnlund A, de Jong TD, Betteridge Z, McHugh N, et al. Autoantibody Specificities and Type I Interferon Pathway Activation in Idiopathic Inflammatory Myopathies. Scand J Immunol. 2016 Aug;84(2):100–9.

5. Maria NI, van Helden-Meeuwsen CG, Brkic Z, Paulissen SMJ, Steenwijk EC, Dalm VA, et al. Association of Increased Treg Cell Levels With Elevated Indoleamine 2,3-Dioxygenase Activity and an Imbalanced Kynurenine Pathway in Interferon-Positive.

6. Maria NI, Steenwijk EC, IJpma AS, van Helden-Meeuwsen CG, Vogelsang P, Beumer W, et al. Contrasting expression pattern of RNA-sensing receptors TLR7, RIG-I and MDA5 in interferon-positive and interferon-negative patients with primary Sjögren’s syndrome. Ann Rheum Dis. 2017 Apr;76(4):721–30.

7. Merrill JT, Immermann F, Whitley M, Zhou T, Hill A, O’Toole M, et al. The Biomarkers of Lupus Disease Study: A Bold Approach May Mitigate Interference of Background Immunosuppressants in Clinical Trials. Arthritis Rheumatol (Hoboken, NJ). 2017 Jun;69(6):1257–66.

8. Wither J, Johnson SR, Liu T, Noamani B, Bonilla D, Lisnevskaia L, et al. Presence of an interferon signature in individuals who are anti-nuclear antibody positive lacking a systemic autoimmune rheumatic disease diagnosis. Arthritis Res Ther. 2017 Feb;19(1):41.

9. Cooles FAH, Anderson AE, Lendrem DW, Norris J, Pratt AG, Hilkens CMU, et al. The interferon gene signature is increased in patients with early treatment-naive rheumatoid arthritis and predicts a poorer response to initial therapy. Vol. 141, The Journal of allergy and clinical immunology. 2018. p. 445-448.e4.

10. Kamiyama R, Yoshimi R, Takeno M, Iribe Y, Tsukahara T, Kishimoto D, et al. Dysfunction of TRIM21 in interferon signature of systemic lupus erythematosus. Mod Rheumatol. 2018 Nov;28(6):993–1003.

11. Rodríguez-Carrio J, López P, Alperi-López M, Caminal-Montero L, Ballina-García FJ, Suárez A. IRF4 and IRGs Delineate Clinically Relevant Gene Expression Signatures in Systemic Lupus Erythematosus and Rheumatoid Arthritis. Front Immunol. 2018;9:3085.

12. Wahadat MJ, Bodewes ILA, Maria NI, van Helden-Meeuwsen CG, van Dijk-Hummelman A, Steenwijk EC, et al. Type I IFN signature in childhood-onset systemic lupus erythematosus: a conspiracy of DNA- and RNA-sensing receptors? Arthritis Res Ther. 2018 Jan;20(1):4.

13. Blokland SLM, van den Hoogen LL, Leijten EFA, Hartgring SAY, Fritsch R, Kruize AA, et al. Increased expression of Fas on group 2 and 3 innate lymphoid cells is associated with an interferon signature in systemic lupus erythematosus and Sjögren’s syndrome. Rheumatology (Oxford). 2019 Oct;58(10):1740–5.

14. Brohawn PZ, Streicher K, Higgs BW, Morehouse C, Liu H, Illei G, et al. Type I interferon gene signature test-low and -high patients with systemic lupus erythematosus have distinct gene expression signatures. Lupus. 2019 Nov;28(13):1524–33.

15. Lambers WM, de Leeuw K, Doornbos-van der Meer B, Diercks GFH, Bootsma H, Westra J. Interferon score is increased in incomplete systemic lupus erythematosus and correlates with myxovirus-resistance protein A in blood and skin. Arthritis Res Ther. 2019 Dec;21(1):260.

16. Olsson P, Bodewes ILA, Nilsson AM, Turesson C, Jacobsson LTH, Theander E, et al. Associations of cigarette smoking with disease phenotype and type I interferon expression in primary Sjögren’s syndrome. Rheumatol Int. 2019 Sep;39(9):1575–84.

17. Palli E, Kravvariti E, Tektonidou MG. Type I Interferon Signature in Primary Antiphospholipid Syndrome: Clinical and Laboratory Associations. Front Immunol. 2019;10:487.

18. Pescarmona R, Belot A, Villard M, Besson L, Lopez J, Mosnier I, et al. Comparison of RT-qPCR and Nanostring in the measurement of blood interferon response for the diagnosis of type I interferonopathies.

19. Björk A, Richardsdotter Andersson E, Imgenberg-Kreuz J, Thorlacius GE, Mofors J, Syvänen A-C, et al. Protein and DNA methylation-based scores as surrogate markers for interferon system activation in patients with primary Sjögren’s syndrome. RMD open. 2020 Jan;6(1).

20. Houssiau FA, Thanou A, Mazur M, Ramiterre E, Gomez Mora DA, Misterska-Skora M, et al. IFN-α kinoid in systemic lupus erythematosus: results from a phase IIb, randomised, placebo-controlled study. Ann Rheum Dis. 2020 Mar;79(3):347–55.

21. Smith MA, Chiang C-C, Zerrouki K, Rahman S, White WI, Streicher K, et al. Using the circulating proteome to assess type I interferon activity in systemic lupus erythematosus. Sci Rep. 2020 Mar;10(1):4462.

22. Tesser A, de Carvalho LM, Sandrin-Garcia P, Pin A, Pastore S, Taddio A, et al. Higher interferon score and normal complement levels may identify a distinct clinical subset in children with systemic lupus erythematosus. Arthritis Res Ther. 2020 Apr;22(1):91.

23. Mai L, Asaduzzaman A, Noamani B, Fortin PR, Gladman DD, Touma Z, et al. The baseline interferon signature predicts disease severity over the subsequent 5 years in systemic lupus erythematosus. Arthritis Res Ther. 2021 Jan;23(1):29.

24. Ma Y, Wang M, Jia J, Meng J, Teng J, Zhu D, et al. Enhanced type I interferon signature induces neutrophil extracellular traps enriched in mitochondrial DNA in adult-onset Still’s disease. J Autoimmun. 2022 Feb;127:102793.

25. Yiu G, Rasmussen TK, Tsai BL, Diep VK, Haddon DJ, Tsoi J, et al. High Interferon Signature Leads to Increased STAT1/3/5 Phosphorylation in PBMCs From SLE Patients by Single Cell Mass Cytometry. Front Immunol. 2022;13:833636.

26. Vieira M, Régnier P, Maciejewski-Duval A, Le Joncour A, Darasse-Jèze G, Rosenzwajg M, et al. Interferon signature in giant cell arteritis aortitis. Journal of autoimmunity. 2022;127:102796.

27. Batten I, Robinson MW, White A, Walsh C, Fazekas B, Wyse J, et al. Investigation of type I interferon responses in ANCA-associated vasculitis. Scientific Reports. 2021;11(1):8272.

28. Shen M, Duan C, Xie C, Wang H, Li Z, Li B, et al. Identification of key interferon-stimulated genes for indicating the condition of patients with systemic lupus erythematosus. Front Immunol. 2022;13:962393.

29. Amezcua-Guerra LM, Sánchez-Muñoz F, Pichardo-Ontiveros E, González-Ramírez J, Martínez-Martínez LA, Juárez-Vicuña Y. Interferon-alpha regulates expression of lncRNA MALAT1 and interferon-stimulated genes, as well as chemokine production, in primary Sjögren’s syndrome. Clin Exp Rheumatol. 2022 Dec;40(12):2275–82.

30. Rice GI, Melki I, Frémond M-L, Briggs TA, Rodero MP, Kitabayashi N, et al. Assessment of Type I Interferon Signaling in Pediatric Inflammatory Disease. J Clin Immunol. 2017 Feb;37(2):123–32.

31. Bodewes ILA, Huijser E, van Helden-Meeuwsen CG, Tas L, Huizinga R, Dalm VASH, et al. TBK1: A key regulator and potential treatment target for interferon positive Sjögren’s syndrome, systemic lupus erythematosus and systemic sclerosis. J Autoimmun. 2018 Jul;91:97–102.

32. Smith S, Fernando T, Wu PW, Seo J, Ní Gabhann J, Piskareva O, et al. MicroRNA-302d targets

IRF9 to regulate the IFN-induced gene expression in SLE. J Autoimmun. 2017 May;79:105–11.
